# Radioactivity of Honeys from Poland After the Fukushima Accident

**DOI:** 10.1007/s00128-013-1089-1

**Published:** 2013-09-04

**Authors:** Maria H. Borawska, Jacek Kapała, Anna Puścion-Jakubik, Justyna Horembała, Renata Markiewicz-Żukowska

**Affiliations:** 1 Department of Bromatology, Medical University of Bialystok, 15-222 Mickiewicza 2D, Poland; 2 Department of Biophysics, Medical University of Bialystok, 15-089 Mickiewicza 2A, Poland

**Keywords:** Honey, Cesium-137, Potassium-40, Poland

## Abstract

Concentration of radioactive isotopes in honey constitutes an important bioindicator of environmental radiation. One hundred six honey samples were collected from hives and from bottled honey provided by beekeepers from north-eastern Poland in 2010, before the Fukushima accident, and during the two-year period directly following this catastrophe (2011–2012). Cesium-137 (Cs-137) and potassium-40 (K-40) were determined in lime, multifloral, buckwheat, honeydew and other kinds of honey samples. The obtained mean concentrations of Cs-137 and K-40 (Bq kg^−1^) in honey samples were: 1.19 and 32.92 in 2010, 0.90 and 31.13 in 2011, 1.31 and 36.06 in 2012, respectively. Significant differences were not observed. Therefore, the studied honey samples collected after the Fukushima accident are found to be safe for humans with levels of Cs-137 and K-40 not posing any threats. However, the total concentration of Cs-137 and K-40 in samples stopped decreasing in 2010–2011 and showed a slight increase in 2012. This relation may suggest the impact of pollution from Fukushima and requires further research in the coming years.

Products of *Apis mellifera*, such as honey, have been widely used for centuries in traditional medicine all over the world due to their nutritional and medical properties. Honey is not only a natural sweet product. The fact that it contains many biologically active compounds such as enzymes, proteins, amino acids, flavonoids, aromatic acids, vitamins and carbohydrates causes honey to have beneficial effects in the human diet with its antioxidant, anti-bacterial, anti-viral and anti-fungal activities (Al-Waili et al. 2011; Bogdanov et al. 2008; Liu et al. 2013).

Honey can be used as an environmental marker for assessing the presence of heavy metals (Pb, Cd) (Przybyłowski and Wilczyńska 2001). We also monitored the average Cesium-137 (Cs-137) and potassium-40 (K-40) levels in honeybee honey in the 12 years following the Chernobyl accident (Borawska et al. 2000).

Poland is one of the highly developed countries yet it does not possess nuclear power plants (NPP). In general radiation exposure can be caused by the environment resulting from nuclear explosions, radiation accidents and the presence of artificial radionuclides in foodstuffs. The accident at the NPP Fukushima Dai-ichi in Japan on the 11th of March 2011 caused a leak of radioactive substances into the environment. Due to air movement in the atmosphere, the first radioactive substances released from the Fukushima NPP containing traceable amounts of radionuclides of artificial origin arrived in Poland on the 23rd of March 2011 (National Atomic Energy Agency 2012). That document contained information about concentrations of Cs-137 and Cs-134 isotopes in meat, poultry, fish, eggs, vegetables, fruits, cereals and mushrooms, but not in honey.

At present the concentration of Cs-134 in foodstuffs is below the level of 1 ‰ of Cs-137 activity. In our research we have identified Cs-134 below the method’s detection threshold. Therefore Cs-134 has been omitted in the remaining part of this paper. Additionally, doses of ionizing radiation descend from natural radionuclides, of which the most important is K-40.

The aim of this study was to examine the concentration of Cs-137 and K-40 in different kinds of honey samples from north-eastern Poland before and in the two-year period following the Fukushima accident.

## Materials and Methods

A total of 106 honey samples (lime-19, multifloral-27, buckwheat-22, honeydew-9 and other kinds-29) were collected from 2010 to 2012. Honey samples were obtained from individual beekeepers from north-eastern Poland from a clean and ecological Podlasie region, with a low lead concentration in the food derived from this area (Markiewicz et al. 2006).

In the course of our study we have relied on the gamma-ray spectrum analysis method (Currie 1968).

Honey samples were measured in flat 30 cm^3^ vessels. Activity measurements were performed by a coaxial germanium detector having its own thin-window which expands the useful energy range down to 5 keV (relative efficiency 30 % model GX3020) and a computer system for gathering and analyzing spectra, Genie-2000 (CANBERRA). The detector was placed in a shielded house built from low-background, 10 cm thick lead, lined with a sheet of copper (thickness – 1.5 mm) and a sheet of aluminum (0.5 mm thick) to avoid soft X radiation, which occurs as a result of interaction between gamma radiation and lead. This method secures low detection limits, with the use of 30 cm^3^ vessels, counted with Lloyd A. Currie method (Currie 1968) for the 661.67 keV line (Cs-137) and 1460.83 line (K-40) on 0.07–0.19 Bq kg^−1^ level for Cs-137 and 2–3.5 Bq kg^−1^ level for K-40, respectively. The samples were examined for 70 h. The measurements were carried out at the Department of Biophysics at the Medical University of Bialystok, which constitutes one of the 13 automatic Permanent Monitoring Stations owned by the National Atomic Energy Agency and operates within the international Framework of the European Union (EU) and Baltic states (the Council of Baltic Sea States) network.

## Results and Discussion

Labeling of honey must be supported by analysis that confirms its provenance and safety. The EU is the world`s largest consumer of honey (Vanhanen et al. 2011) and the need of assuring low levels of radionuclides in honey is very high given the constantly rising global trends in honey production.

In our honey samples concentrations of Cs-137 ranged from 0.16 to 6.38 Bq kg^−1^ in 2010, before the Fukushima accident, and from 0.11 (lime honey) to 16.39 (the other honey) Bq kg^−1^ in 2011 and 2012 – following the Fukushima accident (Table 1). The notably lowest contents of Cs-137 were in lime honey in 2011, with a mean of 0.35 Bq kg^−1^ and 2012, with a mean of 0.51 Bq kg^−1^, while in honeydew honey samples they were slightly higher than in 2010. A higher variability in radioactive Cs-137 content in samples of honey is related to the period of harvesting and hive placement in a given year.

**Table 1:** Content of Cs-137 and K-40 in natural bee honey samples

No	Type of honey	n			Cs-137 (Bq kg^−1^)			K-40 (Bq kg^−1^)		
					Mean ± SD			Mean ± SD		
					(Min–Max)			(Min–Max)		
		2010	2011	2012	2010 (a)	2011 (b)	2012 (c)	2010 (d)	2011 (e)	2012 (f)
1	Lime honey	5	7	7	2.20 ± 2.6*	0.35 ± 0.2	0.51 ± 0.5	32.87 ± 14.6^#^	30.91 ± 11.8	57.98 ± 13.4^##^
					(0.25–6.38)	(0.11–0.64)	(0.13–1.56)	(17.42–49.44)	(12.63–49.6)	(35.17–78.40)
2	Multifloral honey	9	11	7	1.72 ± 1.8	1.54 ± 3.1	0.63 ± 0.6	34.08 ± 19.3	31.40 ± 9.5	23.85 ± 12.3
					(0.16–4.54)	(0.24–10.57)	(0.13–1.99)	(16.90–77.15)	(10.90–43.49)	(10.56–47.63)
3	Buckwheat honey	8	8	6	0.41 ± 0.3	0.39 ± 0.3	0.50 ± 0.7	25.92 ± 6.1	25.25 ± 9.4	28.31 ± 4.8
					(0.25–1.06)	(0.19–0.95)	(0.13–1.88)	(16.34–35.73)	(12.02–43.29)	(20.97–35.92)
4	Honeydew honey	2	3	4	0.22 ± 0.0	1.53 ± 2.3	1.21 ± 1.2	51.46 ± 0.5	53.11 ± 31.0	39.44 ± 23.8
					(0.18–0.25)	(0.15–4.18)	(0.35–2.86)	(51.11–51.80)	(27.14–87.48)	(11.42–69.53)
5	Other honey	3	13	13	0.65 ± 0.8	0.82 ± 0.8	2.52 ± 4.8	35.82 ± 9.2	29.55 ± 9.4	33.37 ± 25.1
					(0.19–1.55)	(0.16–2.84)	(0.13–16.39)	(25.83–43.99)	(12.26–42.59)	(5.51–98.89)
	Total	27	42	37	1.19 ± 1.6	0.90 ± 1.7	1.31 ± 3.0	32.92 ± 14.4	31.13 ± 13.2	36.06 ± 21.3
					(0.16–6.38)	(0.11–10.57)	(0.13–16.39)	(16.34–77.15)	(10.90–87.48)	(5.51–98.89)

n – quantity of samples; *SD* standard deviation

Level of significance: * p_a/b+c_ < 0.02, ^#^ *p*_d/f_ < 0.02, ^##^ *p*_e/f_ < 0.002

The obtained data indicates that in 2011 and 2012 average activity of cesium isotope in honey was at the same level as in the previous year, i.e. – 2010. Compared to year 1998 (12 years following the Chernobyl accident), the activities were several (3–5) times lower (Borawska et al. 2000) in 2011–2012 and hence, 1 and 2 years following the Fukushima accident. Moreover, the data provided by The Polish National Atomic Energy Agency indicates that in 2011 the average activity of cesium isotope in meat, poultry, fish and eggs was on the same level as in the previous year (National Atomic Energy Agency 2012).

The mean concentration of K-40 in honey samples was 32.92 Bq kg^−1^ in 2010 and did not significantly change in 2011–2012. Compared to year 1998 – 12 years after the Chernobyl accident – it was more than two times lower (Borawska et al. 2000). The increased content of Cs-137 in lime honey harvested in 2010 summer may be due to surface soil re-suspension of the radionuclide. Open burning of grass is a common practice in Poland. Extensive forest fires, especially in the areas contaminated by radioactivity, might have contributed to the increase of concentration of radioactive elements (Yoschenko et al. 2006). In 2010, the fires took place in Russia. This suggests that, as a result of fires, a particle of matter could be moving at hundreds of kilometers and there could be an increase in background radiation (Charles 2010 and Jargin 2011). However, researchers do not agree on the extent to which the fires and winds can affect the spread of these pollutants (Jargin 2011). Jargin (2011) states that forest fires in the vicinity of Chernobyl and Russia do not carry significant risk of radiocontamination. The dominant source of Cs-137 in the air is re-suspension (e.g. due to agricultural activities) of previously deposited Cs-137 in soil and its subsequent transport by winds and precipitation (Matisoff et al. 2011, Povinec et al. 2012, Sýkora et al. 2012). Lower Cs-137 concentration in food may be caused by drought. The total rainfall in Podlasie region in May and June 2011, was 1.7 times lower than in the same months in 2010 – it can explain – higher content of Cs-137 in lime honey from 2010 (National Atomic Energy Agency 2012 and Central Statistical Office 2012). The average concentration of Cs-137 in Poland, during the period of monitoring of the soil radioactive contamination, decreased from 4.64 kBq/m^2^ in 1988 to 1.93 kBq/m^2^ in 2010. In soil it is 20 times lower than the average content of natural radionuclide K-40 (National Atomic Energy Agency 2012). Comparing to year 1986 (Chernobyl accident), the average activities of Cs-137 in meat, poultry, fish and eggs in 2010 were also several times lower.

National regulations determine annual effective dose limits for population which is set at 1 mSv. However, K-40 was not taken into account for dose calculation, as potassium is an essential element. Daily consumption of about 160 kg of tested honey in 2012 causes the annual effective dose limit to be exceeded (International Atomic Energy Agency safety standards series 1996). Fukushima accident did not have any significant influence on the radioactivity of honeys from Poland because concentrations of Cs-137 and K-40 in the studied honeys related to the Fukushima leak were stated to be low and not posing any threats or hazards to the safety of human health. However, the total concentration of Cs-137 and K-40 in honey samples stopped decreasing in 2010–2011 and showed a slight increase in 2012. This relation may suggest the impact of pollution from Fukushima and requires further research in the coming years.
